# The Cross-Sectional Association of Scales from the Job Content Questionnaire 2 (JCQ 2.0) with Burnout and Affective Commitment Among German Employees

**DOI:** 10.3390/ijerph22030386

**Published:** 2025-03-06

**Authors:** Maren Formazin, Peter Martus, Hermann Burr, Anne Pohrt, BongKyoo Choi, Robert Karasek

**Affiliations:** 1 Federal Institute for Occupational Safety and Health (BAuA), Division “Work and Health”, 10317 Berlin, Germany; 2 Institute for Clinical Epidemiology and Applied Biometrics, University Hospital Tübingen, 72076 Tübingen, Germany; peter.martus@med.uni-tuebingen.de; 3 Institute of Biometry and Clinical Epidemiology, Charité—Universitätsmedizin Berlin, Corporate Member of Freie Universität Berlin and Humboldt-Universität zu Berlin, 10117 Berlin, Germany; 4 Center for Work and Health Research, Irvine, CA 92620, USA; 5 Department of Medicine, University of California, Irvine, CA 92617, USA; 6 Department of Work Environment, University of Massachusetts Lowell, Lowell, MA 01854, USA; 7 Department of Psychology, Copenhagen University, Denmark & Øresund Synergy, 1353 Copenhagen, Denmark

**Keywords:** demand–control–support model, task level, organizational level, added variance, multiple imputation, validation sample

## Abstract

The Job Content Questionnaire JCQ 2.0 (JCQ 2.0) thoroughly revises the well-known JCQ 1, based on an expanded Demand/Control theory-consistent platform with new scales, the Associationalist Demand Control (ADC) theory. This study tests the JCQ 2.0 in an urban population in Germany (*N* = 2326) for concurrent validity of each specific task and organizational-level scale and the relative importance of the task and organizational-level scales, using burnout and commitment as outcome measures. Cross-sectional regression analyses in the test and validation samples were run after multiple imputation. Five JCQ 2.0 task-level scales explain 44% of burnout variance; three JCQ 2.0 task-level scales explain 25% of commitment variance. Adding organizational-level scales, organizational disorder and rewards, increases the explained variance for burnout by five percentage points; consideration of workers’ interests and reward add four percentage points of variance for commitment. Organizational-level scales alone explain 33% and 28% of the variance in burnout and commitment, respectively, due to three and five organizational-level scales for both outcomes. Thus, the JCQ 2.0 task and organizational-level scales show substantial relations to work- and health-related outcomes, with task level more relevant for burnout and organizational level more relevant for commitment. The most strongly related JCQ 2.0 scales have evolved from new ADC theory, confirming its utility.

## 1. Introduction

### 1.1. The Demand–Control Model, the Demand–Control–Support Model, and the JCQ 1

Karasek’s job demand–control model (DC model [1]), originally presented in the late 1970s, is a well-established model that has been used in a plethora of studies on psychosocial working conditions and health [2,3,4,5]. The model is built on two underlying dimensions of working conditions, demand and control [1,6]. Demands in the DC model comprise work pace, amount of work, and role conflict. All these can be considered as stressors. The second dimension, control—also labelled decision latitude—comprises two aspects: skill discretion, i.e., the way in which the workers’ abilities are used and developed, and decision authority, i.e., how workers can influence their work. Consequently, decision latitude/control can be considered a resource that helps workers to attain their goals and is supposed to buffer the negative effects of demands. Both dimensions—demand and control—are deemed independent of one another [1,6]. Among the four possible combinations of dichotomized demand and control (i.e., high vs. low for both dimensions), it is the combination of high demand and low control, labelled “job strain”, that is assumed to be especially detrimental to health and work-related outcomes.

Over the years, the original DC model has been tested in a multitude of studies for cardiovascular disease/coronary heart disease [2,3,5], reduced psychological well-being [7], and depression [4].

In the 1980s, Johnson and colleagues [8,9] proposed widening the original DC model with a third dimension: social support. This dimension is again described as independent of the two others and comprises social support by co-workers on the one hand and social support by the supervisor on the other hand.

The three dimensions of the demand–control–support model (DCS model) and their sub-dimensions—as described above—can be assessed with the “Job Content Questionnaire” with the latest edition JCQ 1.7 dating from 1996, with primary scales defined in the JCQ 1.1 as quantitative demands, skill discretion, decision authority, supervisor support, and co-worker support (hereafter referred to as the JCQ 1 [10]). The JCQ 1 is, however, not confined to the DCS model and assesses additional aspects of the work environment, e.g., physical demands, job insecurity with a focus on losing one’s job, and job insecurity referring to aspects of skill obsolescence [11].

### 1.2. Updating the JCQ for the Modern Work Reality

The scales in the JCQ 1 mainly refer to work characteristics at the task level, i.e., the workers’ specific work task and their direct contacts in the work environment, as they were present in industrialized economies among large companies. However, since the 1980s, the world of work has changed, e.g., due to the growth of the service economy, digitalization, and globalization. Consequently, the JCQ 1 in its version from 1985 does not assess all work characteristics at the task level deemed relevant in today’s world of work for a large number of workers. For example, emotional demands at work are relevant for health [12,13,14] but have not been assessed with the JCQ in the past. Similarly, negative acts (e.g., bullying), which nowadays are established as antecedents to health outcomes, are missing as well.

Moreover, workers are exposed to additional working conditions to those from the task level, namely those stemming from the organizational level. These are not assessed by the JCQ 1 either, even though the organization at least partly determines how processes at the task level are designed [A.1]. Examples of missing aspects are organizational justice, climate (i.e., psychosocial safety climate), and (dis-)order. Knowledge on organizational work characteristics is essential in order to derive effective interventions for improving the work environment as these influence health and work-related outcomes, either directly or indirectly via the task level [15].

Furthermore, the organization is embedded in the labour market and hence subject to external influences, i.e., influences from the “external-to-work” level [A.1]. Globalization and digitalization in the labour market influence the organization, which in turn influences the structure of work at the task level.

### 1.3. Revising the JCQ

Because the JCQ 1 lacks scales assessing these different aspects [11], it became necessary to enhance the available tool. The new version is based on the enhanced theoretical framework, the “Associationalist D/C Model” (ADC model [A.1]).

At the same time, it was deemed necessary to shorten the existing scales to a minimum set of relevant items, nevertheless retaining reliable and valid measurements, in order to avoid an overly lengthy questionnaire that would pose a burden to employees who fill it out.

The development of the JCQ 2.0 was undertaken by the JCQ expert group, a collaboration of experts on work stress from different parts of the world (Europe, Asia, USA, and Australia) with experience on JCQ usage (e.g., R. Karasek, S. Cho, B. Choi, M. Dollard, I. Houtman, W. Agbenyikey, J. Li, and M. Formazin). The development process is described in more detail by Agbenyikey et al. [A.2]. The group’s aim was to revise and enhance the available JCQ 1 tool considering the following points:

- Shortening and partly revising the available task-level scales from JCQ 1 by replacing single items while ensuring that scales remain reliable and valid;
- Enhancing the assessment of work characteristics at the task level with additional scales that capture aspects of the work environment that have gained relevance in the past;
- Complementing the JCQ instrument with organizational-level scales that cover demand, control, and support according to the ADC model.

The process of arriving at the revised JCQ 2.0 as well as the empirical results of the multiple steps that were carried out are described in detail in Agbenyikey et al. [A.2].

### 1.4. The JCQ 2.0 Task- and Organizational-Level Scales

For this paper, 18 scales with 54 items from the JCQ 2.0 are considered: ten scales at the task level and eight at the organizational level, following the basic principle of the ADC model with three distinguishable dimensions for demand, control, and support. Table 1 gives an overview of the scales incorporated into the JCQ 2.0 as considered in this study. Among the eighteen scales, six were available in the JCQ 1 already and twelve scales were newly added (italic font in Table 1). Beyond this paper, the JCQ 2.0 also includes scales to assess external-to-work factors, such as expanded job insecurity and work-family challenges [A.3].

### 1.5. Scales for the Task Level

In this and the following section, a short description of the scales from the JCQ 2.0 used in this paper will be presented. A detailed description of these scales is presented by Karasek and colleagues [A.1].

#### 1.5.1. Demands

At the task level, three scales refer to demands. Two scales, “physical demands” and “quantitative demands”, were already part of the JCQ 1 (see Section 1.1), with the latter scale labelled “psychological demands” then. To avoid ambiguity, this was changed for the JCQ 2.0 and the scale was shortened. The third scale, “emotional demands”, is new to the JCQ 2.0 and refers to the requirement on workers to regulate their feelings and expressions for organizational goals when dealing with other people, i.e., customers or clients, at work [13].

#### 1.5.2. Control

The JCQ 2.0 contains three task-level control scales. Two scales, “decision authority” and “skill discretion”, were taken from the JCQ 1 with the latter scale now shortened. A third scale, “conducive development”, was newly added to the JCQ 2.0 and focuses on the continuous development of abilities [16].

#### 1.5.3. Stability-Support (Support Is Re-Labelled in the ADC Model)

In the JCQ 2.0, there are four scales available to assess social support at the task level. The two established scales from the JCQ 1, namely “support by supervisor” and “support by co-workers”, were kept and revised, now also incorporating the aspect of respect; the latter scale is conceptualized as “people one works with”, which is wider than just colleagues (e.g., including suppliers or business partners). Moreover, two scales were newly added to the JCQ 2.0: The scale “collective control” refers to collective forms of control among colleagues through the development of work groups [17] deemed protective for workers’ health [18]. The scale “negative acts” was added because such behaviour is associated with workers’ mental health problems cross-sectionally [19] and longitudinally [4], albeit differentially for men and women [20].

Nearly all established JCQ scales (except decision authority) were shortened compared to the version available in the JCQ 1, and the phrasing of some items was adapted. A combination of criteria for shortening and reformulating the scales was applied: it was deemed necessary to retain items (i) with substantial corrected item–total correlations, (ii) with clarity of content, and (iii) with high comprehensibility for a wide range of workers. At the same time, the correlations of JCQ scales with other relevant measures, e.g., burnout and commitment, were supposed to at least remain the same or increase. Further scales available in the JCQ 1, albeit not part of the DCS model (e.g., “physical demands”) were revised, too. A thorough description of the revision process can be found in Agbenyikey et al. [A.2].

### 1.6. Scales for the Organizational Level

#### 1.6.1. Demands

For organizational demands, two new scales were developed: “Organizational disorder”, e.g., badly organized processes in an organization, is associated with extra load on the workers that they consider unnecessary. In addition, a scale assessing “restructuring” [21,22,23,24] was developed.

#### 1.6.2. Control

Control at the organizational level is assessed with three scales: The scale “organizational decision latitude” describes to what extent workers are consulted in matters that will affect them [25]; “procedural justice” describes the extent to which decision-making procedures are fair (i.e., with input from affected parties, suppressing bias; summarized in [26]); and “conducive communication” captures aspects of innovation brought about by co-partnering between employees and customers and by a focus on customer needs for production [16] and is an important scale for future use of the JCQ2. In Karasek et al. [A.1], we recommend several additional questions for this scale’s coming use to even better capture the construct.

#### 1.6.3. Stability-Support

The dimension of support at the organizational level comprises three aspects: “consideration of workers’ interests”, “psychosocial safety climate (PSC)”, and “rewards”. PSC is a facet-specific dimension of organizational climate referring to shared perceptions regarding “policies, practices, and procedures for the protection of worker psychological health and safety” [27]; p. 579. The concept of “rewards” comprising—among other aspects—recognition and financial rewards is based on Siegrist’s effort–reward imbalance (ERI) model [28,29].

### 1.7. Aims of This Paper

The reliability of the new JCQ 2.0 scales has been tested [A.2]; empirical support for the three-factor structure of demand, control, and stability-support at the task and organizational levels has been presented [A.4]; and associations of the three factors at each level with relevant outcome measures have been shown [A.5], providing support for continued usage of the D/C/S-S scale structure narrative as stated in [A.1]. However, little is known about exactly which scales conceptualized at the task or organizational level are the specifically relevant predictors for health and work-related outcomes. Yet, such knowledge is necessary to ensure that risk factors in the psychosocial work environment are not overlooked due to different risk factors being merged into one score for each factor [30]. It also more directly connects JCQ 2.0 testing to the existing literature in the field. Thus, the focus of this paper is on the JCQ 2.0 scales and how they are related to burnout and affective commitment.

Burnout is conceptualized as a state of exhaustion and fatigue that is attributed to work factors: “the degree of physical and psychological fatigue and exhaustion that is perceived by the person as related to his/her work” [31]; p. 197. It is, among other aspects, prospectively associated with sickness absence (spells and days; [32]) and hence a negative work-related outcome measure for both employees and employers and can be considered as a sign of psychological strain according to the DC model [6]. Affective commitment is conceptualized as a psychological state of “affective or emotional attachment the organization” [33]; p. 2, i.e., how strongly an individual identifies with their organization. High commitment is related to low employee turnover and high job performance as well as organizational citizenship behaviour [34], rendering it a desirable goal for employers and a suitable positive work-related outcome measure, and can be considered as an indicator of the motivational pathway in the DC model [6].

This paper aims at answering the following research questions:

1. Which JCQ 2.0 scales at the task level most strongly explain variance in burnout and commitment?
2. Does the enhancement of the task-level scales with the organizational-level scales explain more variance in burnout and commitment, indicated by at least a small effect, compared to solely the comprehensive task-level scales? If so, which organizational-level scales are the most relevant? If this is the case, it shows the usefulness of the JCQ 2.0 with its new scales at the organizational level.
3. How much variance in burnout and commitment can be explained when solely considering organizational-level scales? Which ones are the relevant scales?

## 2. Materials and Methods

Items for the JCQ 2.0 have consecutively been developed, added, and tested in three earlier pilots in Korea [35], China [36], and Australia [37]; for details, please refer to [A.2]. The analyses presented in this paper are based on a set of 18 JCQ 2.0 scales, applied in a sample in Germany.

### 2.1. Translation and Pre-Test

The full set of English JCQ 2.0 items was translated via a forward–backward procedure with two German native speakers responsible for forward translation and two English native speakers responsible for backward translation [38,39]. In each step, consensus between both translators was sought. Additionally, after forward and backward translation, inconsistencies were discussed among the members of the JCQ expert group.

To ensure comprehensibility of the translated German items, they were pre-tested among 94 public service employees of a federal German institute, leading to further adaptions of item formulations. Again, these adaptions were agreed upon in the JCQ expert group.

### 2.2. Population

Data were collected by means of a postal survey among the inhabitants of Bochum, a city in the western part of Germany with a diverse economic structure, ranging from production/industry to service and higher education. This large diversity in the working population from production workers to service workers was chosen to ensure that the JCQ 2.0 was tested in a diverse population. Examples include workers from the metal industry, mechanics, physiotherapists, and secondary education teachers (to name just a few). A random sample of 18-to-65-year-old individuals was drawn from a registry office and invited to participate in the study. The registry data solely contained information on age, gender, and address but no information on working status. Consequently, the group of selected individuals also included unemployed or retired people as well as, e.g., students or people on maternity/paternity leave. Hence, in order to reach a sufficient number of employees among those selected, a large sample of 15,000 people was contacted. The required sample size to identify correlations between two variables indicating a small effect of *r* = 0.10 with a power of (1 − β) = 0.80 and α = 0.01 (one-sided) is *n_required_* = 998 [40].

In order to enhance participation among selected persons, they were contacted three times with an invitation letter to the study (supplemented with a letter from the city’s mayor supporting the study), the questionnaire, and a reminder letter. In addition, the study was presented at the city’s weekly press conference, an interview with the project leader in Germany (MF) was shown on local television, and a telephone hotline for interested participants was established.

From the 15,000 questionnaires sent out, 3567 were returned; hence, the response rate was 23.8% among all those contacted. For further analyses, 991 questionnaires had to be excluded either because respondents were not working (retired, students, unemployed, or on maternity/paternity leave) but had received the questionnaire due to the sampling procedure or because they were self-employed/freelancing workers and could not answer items referring to organizational processes in a meaningful way. Additionally, 250 questionnaires from participants with contradictory answers on items referring to the existence of supervisor and/or co-workers and the actual answers given were excluded. In the final analyses, data from 2326 employees was included. Comparisons with available register information on the working population living in Bochum (“Mikrozensus”; https://www.destatis.de/DE/ZahlenFakten/GesellschaftStaat/Bevoelkerung/Mikrozensus.html, accessed on 15 January 2013) implied that response was lower among younger employees, among those with lower levels of education, and among male workers, whereas the response was higher among women, among those with a higher education level, and among those older than 45. A description of the sample can be found in Table 2.

### 2.3. Instruments

The JCQ 2.0 items were to be answered on a four-point Likert scale ranging from “strongly disagree” to “strongly agree” in order to ensure continuity with the JCQ 1 [10] that uses the same answering format. For the following analyses, only a subset of 54 items was used—those that constitute the JCQ 2.0 researcher version of each of the scales considered in this study; the full JCQ 2.0 is presented by Agbenyikey et al. [A.2].

Table 1 gives examples of items, the number of items belonging to each scale, and the scales’ internal consistency for all 18 scales in the German study, which was the last of four international studies [A.2]. For two scales, emotional demands and restructuring, the full researcher scale from the JCQ 2.0 comprises one and two additional items, respectively, that were, however, not available in the German study presented here. Information on the internal consistency of the JCQ 2.0 scales in the other pilot studies is presented in Agbenyikey et al. [A.2].

The majority of the scales fulfil the minimum Cronbach’s Alpha of α = 0.7, indicating acceptable internal consistency [41]. Scales with α < 0.7 are mainly those that comprise a small number of items.

In addition to the JCQ items, respondents answered items on sociodemographic information: age, gender, education, vocational training, and general information about the job (e.g., full-time vs. part-time, years of work experience) and the organization (e.g., number of employees). Furthermore, participants were asked to answer questions on health- and work-related outcomes, two of which will be presented in this paper: burnout and affective commitment.

Burnout was assessed using the scale “work-related burnout” from the Copenhagen Burnout Inventory [CBI], a well-established tool based on a profound development process [31], comprising seven items rated on a five-point scale from “never/almost never” or “to a very low degree” to “always” or “to a very high degree”. An example item is “Do you feel worn out at the end of the working day?” [31]. In our sample, Cronbach’s Alpha for the scale is α = 0.89.

Commitment was assessed using the scale “affective commitment” from a well-established conceptualization of commitment [33,42] with four items rated on a five-point scale from “strongly disagree” to “strongly agree”. An example item is “My organisation has a great deal of personal meaning for me” [33]. In our sample, Cronbach’s Alpha for the scale is α = 0.82.

### 2.4. Handling Missing Data

Over all JCQ 2.0 items, 2.1% of answers were missing, ranging between 0.2% for an item belonging to the scale co-worker support and 5.2% for an item assessing procedural justice. Confining analyses to employees with complete data on the JCQ items would have reduced the sample to *n_complete_* = 1651 (71%). Ignoring missing data leads to biased estimates if the data are not missing completely at random (MCAR) and reduces statistical power [43]. Missing data in the JCQ 2.0, as well as in the outcome measures, were considered to be “missing at random (MAR)” [44], implying that missingness can depend on the missing values but is independent after conditioning on other available data [45]. Multiple imputation is an appropriate method for handling MAR data, allowing it to be properly accounted for in later analyses [46,47].

For the JCQ 2.0 items, *m* = 10 filled-in datasets were generated, incorporating sociodemographic information (age, gender, living with a partner, living with children, information on completed education, years of work experience, job position, occupation, shift work, full-time/part-time, and limited/unlimited contract) in the imputation model. This was done because logistic regression had indicated that missingness in JCQ 2.0 items was not independent of sociodemographic information. “Fully conditional specification (FCS)” was applied, which is appropriate for randomly scattered missing data points [48]. Constraints for minimum and maximum values were set for the metric variables in order to obtain plausible imputed values.

For burnout and commitment, 0.8% and 0.7% of answers were missing, respectively. To reduce model complexity, imputation for outcome measures was conducted separately and followed a two-step procedure. In step 1, separately for each scale, the items belonging to this scale were imputed based on observed values for items from the same scale with gender and age as additional variables in the model. Subsequently, single-item outcome measures and scales for which no values could be imputed in step 1 were imputed simultaneously, again with gender and age as additional variables in the model. In accordance with imputation for the JCQ 2.0 items, *m* = 10 filled-in datasets were generated.

### 2.5. Analyses

Because the majority of scales from the JCQ 1 had been revised for the JCQ 2.0, the formulas to compute scale scores as sum scores according to the JCQ User Guide for JCQ 1.7 [10] could not be applied. Instead, all JCQ scale scores were computed as mean scores over all available items belonging to the respective scales as is done for other questionnaires, e.g., the Copenhagen Psychosocial Questionnaire COPSOQ; [49] or the CBI [31] after reverse-coding of items formulated in a negative way. For users who need to benchmark their scale scores with the JCQ 1, an “archival version” JCQ 2.0 is available with several questions re-added in accordance with the original User’s Guide 1.7. Intercorrelations between JCQ scale scores ranged between |*r*| = 0.05 (skill discretion and restructuring) and |*r*| = 0.66 (organizational decision latitude and procedural justice); the full table of scale intercorrelations is presented in Agbenyikey et al. [A.2].

Scale scores for burnout and commitment were determined as mean scores, too. For all scales, a high score indicates a high value in the underlying dimension.

For regression analyses, all JCQ scale scores as well as burnout and commitment scores and age were centred.

**Figure 1:**
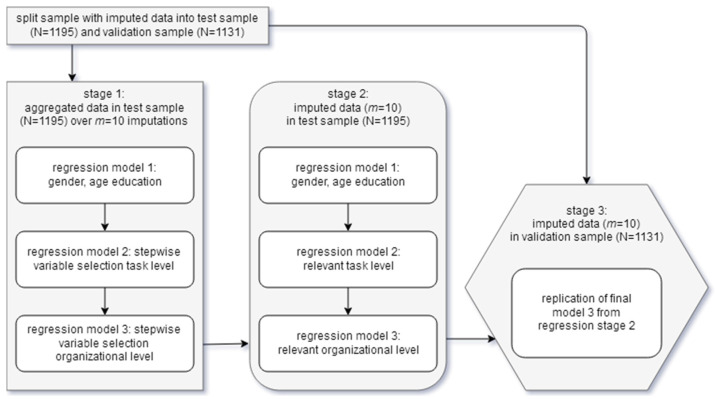
Process of regression analyses—added variance of organizational-level scores over task-level scores.

The analyses procedure is graphically described in Figure 1. The complete dataset was randomly divided into a test sample and a validation sample of approximately equal size to allow for the derivation of a regression model in the test sample and its validation in the validation sample, leading to unbiased estimates for *R*^2^.

Regression analyses followed a three-stage procedure. In the first stage of variable selection, the centred scores were aggregated over the ten imputations (left box, Figure 1). This was carried out to avoid contradictory results over the *m* datasets with stepwise variable selection. The latter can occur with imputed data if predictors have similar beta coefficients. However, because results from this first stage based on aggregated data can only be considered proxies, the model was replicated on the imputed data of the test sample in a second stage to ensure stability of results (middle box, Figure 1). In a third stage, the results were validated on the validation sample (right hexagonal box, Figure 1).

In stage 1, age, gender, and highest school degree—the sociodemographic information considered in the models—were first included in model 1 (first small box in the left box, Figure 1).

The ten task-level scores were then added in a stepwise manner in model 2 (Figure 1, center small box in left box). Variable selection was stopped when the additional explained variance was less than Δ*R_adj_*^2^ = 0.01 in order to consider practical relevance in addition to statistical relevance. Finally, for the third model, the organizational-level scores were added in a stepwise manner, again applying the cut-off value of Δ*R_adj_*^2^ = 0.01 (last small box in the left box, Figure 1). Analyses were then replicated in this way based on the imputed data of the test sample (stage 2, middle box, Figure 1), including age, gender, and highest school degree in model 1; the task-level scale scores identified as significant and relevant in the analyses on aggregated data in model 2 (all added at the same time); and similarly, the relevant organizational-level scale scores identified in model 3 (again, added at the same time). The remaining JCQ scales were then separately tested as possible additional variables.

The final (third) regression model from stage 2 was then applied to the validation sample to allow for a more precise estimation of variance explained by the model by estimating the coefficient of determination *R_adj_*^2^ (stage 3, right hexagonal box, Figure 1). In order to assess the increment in model 3 over model 2 in the validation sample, the effect size *f*^2^ for the increment in explained variance was estimated: based on Cohen’s definition of effect sizes for coefficients of determination *f*^2^, a small effect for the increment in explained variance is a value of *f*^2^ ≥ 0.02, with *f*^2^ ≥ 0.15 indicating a medium effect and *f*^2^ ≥ 0.35 indicating a large effect [50]. We will speak of “incremental” effect size when we refer to *f*^2^.

Because the order in which variables are added to the model determines how much additional variance they can explain, the amount of variance solely explained by the eight organizational-level scales after consideration of age, gender, and highest school degree was additionally estimated in a separate model in order to answer research question 3.

Multiple imputation was run with SPSS 18 [51]; regression analyses were run with SPSS 21 [52].

## 3. Results

### 3.1. JCQ 2.0 Scales and Burnout

In the first model, age, gender, and highest school degree were entered into the regression model for burnout based on the aggregated data of the test sample (stage 1), leading to a small amount of explained variance (model 1, left box in Figure 1). In model 2, among the ten JCQ task-level scores, five emerged as relevant for burnout: quantitative demands, followed by collective control, emotional demands, conducive development, and negative acts (model 2, left box in Figure 1). All associations were in the expected direction, i.e., positive for quantitative and emotional demands and negative acts and negative for collective control and conducive development. When this model was replicated on the imputed data of the test sample, the explained variance in burnout was in the range 0.45 ≤ *R_adj_*^2^ ≤ 0.46 (model 2 in centre box in Figure 1). No further JCQ task-level scale explained additional variance.

Stepwise variable selection for the JCQ organizational-level scores was then applied on the aggregated data of the test sample while considering the five JCQ task-level scores plus the three sociodemographic variables to derive model 3 (in the left box in Figure 1). Only one organizational-level scale emerged as relevant: organizational disorder. Replication on the imputed data of the test sample (stage 2) indicated that rewards were an additional negative relevant variable in explaining variance in burnout with Δ *R_adj_*^2^ > 0.01 in six out of ten imputations. Hence, rewards were kept in the final model (model 3 in centre box, Figure 1), rendering 0.48 ≤ *R_adj_*^2^ ≤ 0.49.

Based on the validation sample and the final model with five task-level and two organizational-level scales in addition to sociographic information, work characteristics explained 0.48 ≤ *R_adj_*^2^ ≤ 0.49 of the burnout variance (stage 3, right hexagonal box in Figure 1), which is virtually the same result as for the test sample. The effect size *f²* for the increment in model 3 over model 2 in the validation sample—the latter with 0.44 ≤ *R_adj_*^2^ ≤ 0.45—was in the range 0.07 ≤ *f*^2^ ≤ 0.10, indicating a small incremental effect. The results of the regression analyses of burnout on the JCQ task and organizational levels are summarized in Table 3.

To test how much variance in burnout was solely explained by the organizational-level scales, another model was established that only considered these scale scores in addition to sociodemographic information, disregarding the task-level scales. Three scales emerged as relevant in stepwise selection: organizational disorder and rewards—the two that also had incremental validity over and above the JCQ task-level scales—and, additionally, consideration of workers’ interests. The three scales explained 0.32 ≤ *R_adj_*^2^ ≤ 0.34 of the variance in burnout in the validation sample, which is 15.0 to 16.1 percentage points less explained variance than the combination of task- and organizational-level scores and also less explained variance than solely considering the task-level scales (i.e., model 2).

### 3.2. JCQ2.0 Scales and Commitment

In stage 1, again entering gender, age, and highest school degree into the regression model for commitment, based on aggregated data of the test sample, led to a small explanation of variance in the outcome measure (model 1 in left box, Figure 1). In stepwise variable selection of task-level scales, three variables emerged as relevant: supervisor support, followed by conducive development and co-worker support (model 2 in left box, Figure 1). This model replicated on the imputed test data resulted in 0.28 ≤ *R_adj_*^2^ ≤ 0.29 (model 2 in centre box, Figure 1). The three beta coefficients were in the expected direction, i.e., the scales were positively associated with commitment. No other scales at the task level explained additional variance in commitment.

Via additional variable selection of organizational-level scales—while considering the three sociodemographic variables and the three JCQ task-level scales supervisor support, conducive development, and co-worker support—two scales emerged as relevant: consideration of workers’ interests and organizational disorder, the former with a positive and the latter with a negative beta value. Model 3 was again replicated on the imputed data of the test sample, leading to 0.32 ≤ *R_adj_*^2^ ≤ 0.34 (stage 2, centre box in Figure 1). No further organizational-level scales emerged as additionally relevant.

This final model applied to the imputed data of the validation sample led to 0.29 ≤ *R_adj_*^2^ ≤ 0.30 (stage 3, right hexagonal box in Figure 1), indicating that the explained variance in commitment dropped by around three percentage points in the validation sample compared to the test sample. The effect size *f²* for the increment in model 3 over model 2 in the validation sample—the latter with 0.25 ≤ *R_adj_*^2^ ≤ 0.26—was in the range 0.05 ≤ *f*^2^ ≤ 0.06, indicating a small incremental effect. A summary of the results can be found in Table 4.

Again, a separate model was established that disregarded the task-level scales and only used the eight organizational-level scales in addition to sociodemographic information. Five scales emerged as relevant in stepwise selection: organizational disorder and consideration of workers’ interests—the two that also had incremental validity over and above the JCQ task-level scales—and three additional ones: rewards, organizational decision latitude, and conducive communication. The five scales explained 0.28 ≤ *R_adj_*^2^ ≤ 0.29 of the variance in commitment in the validation sample, which is 0.7 to 1.5 percentage points less than the combination of task- and organizational-level scores and more explained variance in commitment than considering the task-level scales alone (i.e., model 2).

## 4. Discussion

The aim of this paper was to show the relation of the JCQ 2.0 scales to two relevant health and work-related outcome measures, burnout and commitment, focusing on the task vs. the organizational level of the scales. In summary, one can state that work characteristics as assessed by the scales in the revised JCQ 2.0 instrument explain a substantial amount of variance in both commitment and—to a larger degree—burnout. It is noteworthy that many of the most strongly associated scales evolved directly out of the new ADC theory, and several others were selected from the field literature for inclusion in the ADC theoretical basis. The results imply that the JCQ 2.0 assesses the work environment—which has undergone profound changes due to the global economy [A.1]—well.

The task-level scales from the JCQ 2.0 allow for a substantial explanation of variance in burnout and commitment, with about 44 and about 25 percent, respectively. In relation to research question 1, it is interesting to note that among the five task-level scales selected as relevant in the explanation of burnout, only one—namely, quantitative demands—stems from the JCQ 1. Here, our study replicates the finding that quantitative demands are substantially related to burnout [53], which is in line with DC theory [6]. The other four relevant predictors for burnout are newly developed JCQ 2.0 scales: collective control, emotional demands, conducive development, and negative acts. Hence, for burnout, scales from all three factors of demand, control, and support at the task level are relevant. For commitment, two of the three task-level scales, supervisor support and co-worker support, stem from JCQ 1 and only one stems from JCQ 2.0, namely conducive development. One can hence state that mainly scales from the task-level factors of support and, additionally, control were relevant in the explanation of variance in commitment. Moreover, only one scale—conducive development, directly derived from ADC theory—emerged as significant for both outcome measures; its relevance is emphasized by Karasek et al. [A.1].

It was shown that organizational-level scales explain additional variance in burnout and commitment over and above task-level scales, raising the proportion of explained variance to about 49 percent (+5 percentage points) and to around 29 percent (+4 percentage points), respectively, due to two additional scales in both cases, affirmatively answering research question 2. For both outcome measures, the incremental effect size was of a small magnitude. Among the organizational-level scales, organizational disorder could explain additional variance in both burnout and commitment, indicating that demands at the organizational level in particular are additionally relevant with regard to relevant work-related outcome measures. Rewards only emerged as relevant for burnout and consideration of workers’ interests only emerged as relevant for commitment. Both scales are indicators of social support at the organizational level, underlining its relevance in relation to work-related outcome measures. The analyses did not identify any control scale at the organizational level as explaining additional variance in burnout and commitment.

With regard to research question 3, when solely considering the organizational-level scales, around 33 percent variation in burnout and about 28 percent variation in commitment could be explained. For both outcome measures, the two scales that had already emerged as additionally relevant in the combined task- and organizational-level model were again marked as relevant: organizational disorder and rewards for burnout; organizational disorder and consideration of workers’ interests for commitment. One additional organizational-level scale for burnout and three additional organizational-level scales for commitment were relevant in this model. Here, an interesting difference between both outcome measures can be observed: while for burnout the explained variance from the organizational-level scales was between 15 and 16 percentage points lower than that from the combined task- and organizational-level model, for commitment the difference in explained variance between both models was very small, with around 1 percentage point. Furthermore, organizational-level scales alone explained a smaller proportion of variance in burnout than task-level scales; the opposite was true for commitment. One can hence reason that task-level scales seem to be of higher relevance for burnout, while this is reversed for commitment, i.e., organizational-level scales are more relevant.

Based on structural equation modelling, it has been shown that the factors underlying demand, control, and support at the organizational level can be considered as antecedents to the factors for demand, control, and support at the task level [A.4]. Hence, the organizational-level scales are assumed to relate to burnout and commitment directly and indirectly through their influence on the task-level scales. Considering these structural aspects, the incremental variance explained by the organizational-level scales in model 3 should be interpreted as the minimum contribution of the organizational-level scales beyond the task scales, while the results of their sole consideration in the model can be considered their maximum contribution. The structural interrelationship between the scales at the task and organizational levels does not allow for estimating the respective contributions of scales from both levels.

Summarizing, the results imply that the JCQ 2.0 scales allow for a substantial explanation of health and work-related outcomes. Both the established revised and the augmented scales on the task level as well as the new scales on the organizational level seem to be relevant in relation to burnout and commitment.

The focus of the analyses presented in this paper was on the JCQ 2.0 scales and their differential relations to burnout and commitment. This was an important step because so far, solely factor scores of demand, control, and support underlying the JCQ 2 have been shown to be related to health and outcome measures [A.5], whereas the scales have not been considered separately. Even though the JCQ 2.0 scales have been shown to conform to a DCS structure as theoretically postulated [A.4], their independent associations with outcome measures are of relevance as they might show differential associations [30] that would point to specific needs for interventions in the design of work. Our analyses revealed that this is indeed the case, especially in relation to the task-level scales where only one scale—conducive development—was substantially related to both burnout and commitment, whereas the other scales were specific to both outcomes, underlining that different working conditions are relevant in relation to different outcome measures. Knowing which specific working conditions are most strongly related to an outcome allows for a much more palpable development of a suitable intervention aimed at promoting health and well-being in the workplace. Our analyses indicate that while interventions to reduce burnout should primarily focus on optimizing quantitative demands, measures to increase supervisor and co-worker support are more likely to increase the workers’ commitment. The specific working conditions as assessed by the JCQ scales with their items can indicate specific intervention measures that are less abstract than the factors of demand, control, and support that are based on aggregated information.

### Strengths and Limitations

The results presented in this paper are based on a large sample of more than 2000 employees randomly drawn from a registry office. Efforts to enhance participation in the study among the selected sample were high; nevertheless, the response rate was low. Compared to the working population living in Bochum, respondents were older and had a higher education level. The response rate among employed women was higher than that among employed men. Hence, elevated associations due to unevenly distributed non-response among the selected persons cannot be ruled out. Still, the participants have a wide range of occupations, covering a wide spectrum of working conditions. This can be considered a strength as this led to high variation in JCQ scores.

Cases with missing information on items were not excluded from the analyses. Instead, multiple imputation was applied to make better use of the incomplete data and properly account for missing information, and all analyses were based on imputed data, leading to more conservative estimates. This becomes obvious when comparing results from complete-case analyses with analyses based on imputed data (the former not presented in the paper): for the former, estimates are higher, indicating overestimation, even though for each item the standard deviation in the imputed data was slightly higher than the one in the original data. We compared the effect of using aggregated data instead of pooled parameters from the imputation sample: this did not affect the results substantially (correlations: median of differences 0.0046, 75% percentile 0.0080, maximum 0.025; only 1 of 153 differences was at least 0.02).

The majority of the tested JCQ 2.0 scales had an acceptable Cronbach’s Alpha of α > 0.7. However, three scales (emotional demands, rewards, and conducive communication) did not meet this value. These scales only comprised a small number of items, namely two to three. Consequently, further efforts to raise the internal consistency of these scales are warranted, e.g., by adding items for scales that are currently very short and only contain a few items.

In order to minimize the effects of spurious associations, the complete sample was split randomly into a test sample and a validation sample, allowing replication of all analyses in the validation sample. This approach led to unbiased estimates, with nearly the same results for *R*^2^ for burnout in the validation sample compared to the test sample, and slightly lower estimates for commitment. Our results imply that the method applied was an appropriate means to protect against overoptimism.

This study is a cross-sectional study and hence does not allow causal inferences to be drawn—the latter was not the aim of the study at hand. Both working conditions and outcome measures were assessed using self-report measures, associated with the risk of common-method bias. However, the design of the study did not allow an assessment of either working conditions or health outcomes through experts.

Another strength of this study is the sophisticated approach to revising the JCQ 2.0 instrument: Five of the six available scales were revised based on empirical information that pointed to items in need of revision [A.2]. Twelve new scales (see Table 1) were derived from theoretical considerations and tested in three earlier pilots in Korea [35], China [36], and Australia [37] prior to being applied in the German sample. The newly developed items were translated using forward–backward translation, and the pre-test ensured that employees understood the items.

## 5. Conclusions

The revised and enhanced JCQ 2.0 with its new scales is a useful tool for broadly assessing the psychosocial work environment. With its 54 items belonging to 18 scales on the task and organizational levels, it allows for an appropriate assessment of the wide spectrum of those working conditions that are substantially related to burnout and commitment. The inclusion of the organizational level in addition to the enhancement of the task level contributes to a substantial explanation of the outcomes and broadens the view of the working environment, allowing for a more precise identification of possible areas for workplace interventions to promote workers’ health and well-being—an aspect that is even further extended in the JCQ 2.0’s external-to-work scales, as reported by Agbenyikey et al. [A.3]. Since many of the most strongly related scales directly evolved from or selected in relation to the new multi-level extension of the Demand/Control model—the ADC theory—this study also confirms the utility of that theoretical extension.

## Figures and Tables

**Table 1:** Scales, example items, number of items, and Cronbach’s Alpha for the JCQ 2.0 scales used in this study.

		Scale with Example Items	Included Items	Cronbach’s Alpha
task level	demand	quantitative demands * (e.g., enough time to get the job done [R])	5	0.72
		*emotional demands* (e.g., suppressing genuine emotion)	2	0.57
		physical demands * (e.g., awkward positions)	5	0.87
	control	skill discretion * (e.g., job creative)	3	0.70
		decision authority (e.g., lot of say)	3	0.76
		*conducive development* (e.g., job motivates to expand skills)	3	0.75
	support	supervisor support * (e.g., supervisor concerned)	3	0.85
		co-worker support * (e.g., people I work with support me)	3	0.82
		*collective control* (e.g., feeling of unity)	3	0.74
		*negative acts* (e.g., harassed/group pressure)	2	0.74
organizational level	de-mand	*organizational disorder* (e.g., work processes disorganized)	4	0.71
		*restructuring* (e.g., frequent management turnover)	1	n. a.
	control	*organizational decision latitude* (e.g., significant influence over decisions)	3	0.70
		*procedural justice* (e.g., hear opinions and concerns)	3	0.83
		*conducive communication* (e.g., customer feedback helps my skills)	3	0.69
	support	*consideration of workers’ interests* (e.g., management insures well-being)	2	0.81
		*psychosocial safety climate* (e.g., good communication and information about psychological health)	4	0.90
		*reward* (e.g., adequate salary)	2	0.62

Notes: n. a. = not available; [R] = item needs to be recoded; * revised for JCQ 2.0 compared to JCQ 1; italic font: newly added scales for JCQ 2.0.

**Table 2:** Description of the German sample for the JCQ 2.0 study.

Sociodemographic Information	Values
age	*Mean* = 45.37 (*SD* = 10.44; *Range* = 19–64)
age—women	*Mean* = 44.75 (*SD* = 10.57; *Range* = 21–64)
age—men	*Mean* = 46.15 (*SD* = 10.23; *Range* = 19–64)
gender	55.4% women, 44.1% men
highest school degree:	
high school degree—grade 12/13	53.3%
degree—grade 10	25.9%
degree—grade 9 or lower	20.2%
highest vocational degree:	
doctoral degree	3.3%
master’s or professional degree from a university	21.7%
master’s or professional degree from a university of applied sciences	11.6%
bachelor degree from a vocational college	16.2%
vocational or technical certificate/diploma from a college	28.2%
vocational or technical certificate/ diploma from a company	14.7%
no vocational degree	2.7%
living with a partner	yes: 76.3%; no: 22.5%
living with a child/children	yes: 42.5%; no: 56.3%
language spoken at home	only German: 95.3%
years of work experience in the current job	*Mean* = 14.99 years (*SD* = 11.58)
working hours per week	
full-time (35 h and more)	72.1%
part-time (15 to 34 h)	23.5%
less than 15 h	3.9%
shift work	
no shift work	59.0%
irregular working hours	23.5%
two shifts	7.6%
three and more shifts	8.0%
employees in the organization	
500 and more employees	53.7%
100 to 499 employees	16.2%
50 to 99 employees	7.4%
20 to 49 employees	6.3%
10 to 19 employees	6.1%
9 or less employees	8.6%
currently smoking	
yes, smoking daily	21.0%
yes, smoking occasionally	6.5%
no, not any more	28.6%
no, never smoked	42.1%

Notes: *N* = 2326; percentages not adding up to 100% are due to missing information and/or rounding errors.

**Table 3:** Results of regression analyses for burnout—added variance of organizational-level scores over task-level scores.

Model	Variables Included	Explained Variance (Stepwise, Test Sample, *N* = 1195)	Explained Variance (Modelwise, Test Sample, *N* = 1195)	Explained Variance (Modelwise, Validation Sample, *N* = 1131)
model 1	gender, age, and highest school degree	0.003 ≤ *R*^2^ ≤ 0.005		*R*^2^ = 0
model 2	model 1 + quantitative demands	0.209 ≤ Δ *R_adj_*^2^ ≤ 0.219	0.45 ≤ *R_adj_*^2^ ≤ 0.46	0.44 ≤ *R_adj_*^2^ ≤ 0.45
	model 1 + collective control	0.111 ≤ Δ *R_adj_*^2^ ≤ 0.118		
	model 1 + emotional demands	0.059 ≤ Δ *R_adj_*^2^ ≤ 0.066		
	model 1 + conducive development	0.041 ≤ Δ *R_adj_*^2^ ≤ 0.048		
	model 1 + negative acts	0.013 ≤ Δ *R_adj_*^2^ ≤ 0.016		
model 3	model 1 + 2 + organizational disorder	0.025 ≤ Δ*R_adj_*^2^ ≤ 0.032	0.48 ≤ *R_adj_*^2^ ≤ 0.49	0.48 ≤ *R_adj_*^2^ ≤ 0.49
	model 1 + 2 + rewards	0.006 ≤ Δ*R_adj_*^2^ ≤ 0.011		

Note: The range of *R*^2^ constitutes the range over the *m* = 10 imputations.

**Table 4:** Results of regression analyses for commitment—added variance of organizational-level scores over task-level scores.

Model	Variables Included	Explained Variance (Stepwise, Test Sample, *N* = 1195)	Explained Variance (Modelwise, Test Sample, *N* = 1195)	Explained Variance (Modelwise, Validation Sample, *N* = 1131)
model 1	gender, age, and highest school degree	0.001 ≤ *R*^2^ ≤ 0.002		0 ≤ *R*^2^ ≤ 0.001
model 2	model 1 + supervisor support	0.205 ≤ Δ *R_adj_*^2^ ≤ 0.213	0.28 ≤ *R_adj_*^2^ ≤ 0.29	0.25 ≤ *R_adj_*^2^ ≤ 0.26
	model 1 + conducive development	0.057 ≤ Δ *R_adj_*^2^ ≤ 0.63		
	model 1 + co-worker support	0.009 ≤ Δ *R_adj_*^2^ ≤ 0.012		
model 3	model 1 + 2 + consideraton of workers‘ interests	0.028 ≤ Δ *R_adj_*^2^ ≤ 0.037	0.32 ≤ *R_adj_*^2^ ≤ 0.34	0.29 ≤ *R_adj_*^2^ ≤ 0.30
	model 1 + 2 + organizational disorder	0.011 ≤ Δ *R_adj_*^2^ ≤ 0.021		

Note: The range of *R*^2^ constitutes the range over the *m* = 10 imputations.

## Data Availability

Due to data privacy regulations, the data cannot be distributed.

## Appendix A. References to Papers in the Special Issue “The Job Content Questionnaire 2.0: A Tool for Measurement of the Psychosocial Work Environment and Sustainable Work Globally”

**A.1** Karasek, R.; Dollard, M.F.; Östergren, P.-O.; Cho, S.-i.; Houtman, I. The Multi-level Job Content Questionnaire 2.0 (JCQ 2.0) and the Associationalist Demand–Control (ADC) Theory for a Sustainable Global Economy. *International Journal of Environmental Research and Public Health* **2025 submitted**.
**A.2** Agbenyikey, W.; Li, J.; Cho, S.-i.; McLinton, S.S.; Dollard, M.F.; Formazin, M.; Choi, B.; Houtman, I.; Karasek, R. International Comparative Reliability and Concurrent Validity Assessment of the Multi-level Job Content Questionnaire (JCQ) 2.0. *International Journal of Environmental Research and Public Health* **2025 submitted**.
**A.3** Agbenyikey, W.; Karasek, R.; Formazin, M.; Cho, S.-i.; Choi, B.; Dollard, M.F.; Li, J.; Houtman, I. Associations of external-to-work scales of the Job Content Questionnaire (JCQ) 2.0 with relevant outcome measures. *International Journal of Environmental Research and Public Health* **submitted**.
**A.4** Formazin, M.; Choi, B.; Dollard, M.; Li, J.; McLinton, S.; Agbenyikey, W.; Cho, S.-i.; Houtman, I.; Karasek, R. The structure of demand, control, and stability-support underlying the Job Content Questionnaire (JCQ) 2.0. *International Journal of Environmental Research and Public Health*** 2025 submitted**.
**A.5** Formazin, M.; Dollard, M.; Choi, B.; Li, J.; Agbenyikey, W.; Cho, S.-i.; Houtman, I.; Karasek, R. International empirical validation and value added of the multilevel Job Content Questionnaire 2.0 (JCQ 2). *International Journal of Environmental Research and Public Health* **2025 submitted**.

## References

[1] Karasek R. (1979). Job demands, job decision latitude, and mental strain: Implications for job redesign. Adm. Sci. Q..

[2] Backé E.M., Seidler A., Latza U., Rossnagel K., Schumann B. (2012). The role of psychosocial stress at work for the development of cardiovascular diseases: A systematic review. Int. Arch. Occup. Environ. Health.

[3] Kivimäki M., Nyberg S.T., Batty G.D., Fransson E.I., Heikkilä K., Alfredsson L., Bjorner J.B., Borritz M., Burr H., Casini A. (2012). Job strain as a risk factor for coronary heart disease: A collaborative meta-analysis of individual participant data. Lancet.

[4] Theorell T., Hammarström A., Aronsson G., Träskman Bendz L., Grape T., Hogstedt C., Marteinsdottir I., Skoog I., Hall C. (2015). A systematic review including meta-analysis of work environment and depressive symptoms. BMC Public Health.

[5] Niedhammer I., Bertrais S., Witt K. (2021). Psychosocial work exposures and health outcomes: A meta-review of 72 literature reviews with meta-analysis. Scand. J. Work Environ. Health.

[6] Karasek R., Theorell T. (1990). Healthy Work: Stress, Productivity and the Reconstruction of Working Life.

[7] van der Doef M., Maes S. (1999). The Job Demand-Control(-Support) model and psychological well-being: A review of 20 years of empirical research. Work Stress.

[8] Johnson J.V., Hall E.M. (1988). Job strain, work place social support, and cardiovascular disease: A cross-sectional study of a random sample of the Swedish working population. Am. J. Public Health.

[9] Johnson J.V., Hall E.M., Theorell T. (1989). Combined effects of job strain and social isolation on cardiovascular disease morbidity and mortality in a random sample of the Swedish male working population. Scand. J. Work Environ. Health.

[10] Karasek R. (1985). Job Content Questionnaire and User’s Guide.

[11] Karasek R., Brisson C., Kawakami N., Houtman I., Bongers P., Amick B. (1998). The Job Content Questionnaire (JCQ): An instrument for internationally comparative assessments of psychosocial job characteristics. J. Occup. Health Psychol..

[12] Diefendorff J.M., Erickson R.J., Grandey A.A., Dahling J.J. (2011). Emotional display rules as work unit norms: A multilevel analysis of emotional labor among nurses. J. Occup. Health Psychol..

[13] Grandey A.A. (2000). Emotion regulation in the workplace: A new way to conceptualize emotional labor. J. Occup. Health Psychol..

[14] Sliter M., Jex S., Wolford K., McInnerney J. (2010). How rude! Emotional labor as a mediator between customer incivility and employee outcomes. J. Occup. Health Psychol..

[15] Morgeson F.P., Dierdorff E.C., Hmurovic J.L. (2010). Work design in situ: Understanding the role of occupational and organizational context. J. Organ. Behav..

[16] Karasek R. (2004). An alternative economic vision for healthy work: Conducive economy. Bull. Sci. Technol. Soc..

[17] Johnson J.V., Johnson J.V., Johansson G. (1991). Collective control: Strategies for survival in the workplace. The Psychosocial Work Environment: Work Organization, Democratization and Health.

[18] Saijo Y., Chiba S., Yoshioka E., Nakagi Y., Ito T., Kitaoka-Higashiguchi K., Yoshida T. (2015). Synergistic interaction between job control and social support at work on depression, burnout, and insomnia among Japanese civil servants. Int. Arch. Occup. Environ. Health.

[19] Warszewska-Makuch M., Bedynska S., Zolnierczyk-Zreda D. (2015). Authentic leadership, social support and their role in workplace bullying and its mental health consequences. Int. J. Occup. Saf. Ergon..

[20] Einarsen S., Nielsen M.B. (2015). Workplace bullying as an antecedent of mental health problems: A five-year prospective and representative study. Int. Arch. Occup. Environ. Health.

[21] Falkenberg H., Fransson E.I., Westerlund H., Head J.A. (2013). Short- and long-term effects of major organisational change on minor psychiatric disorder and self-rated health: Results from the Whitehall II study. Occup. Environ. Med..

[22] Pepper L., Messinger M., Weinberg J., Campbell R. (2003). Downsizing and health at the United States Department of Energy. Am. J. Ind. Med..

[23] Quinlan M. (2007). Organisational restructuring/downsizing, OHS regulation and worker health and wellbeing. Int. J. Law Psychiatry.

[24] Reissmann D.B., Orris P., Roy L., Hartman D.E. (1999). Downsizing, role demands, and job stress. J. Occup. Environ. Med..

[25] Dhondt S., Pot F.D., Kraan K.O. (2014). The importance of organizational level decision latitude for well-being and organizational commitment. Team Perform. Manag..

[26] Bies R.J., Moag J.S. (1986). Interactional Justice: Communication Criteria of Fairness. Res. Negot. Organ..

[27] Dollard M.F., Bakker A.B. (2010). Psychosocial safety climate as a precursor to conducive work environments, psychological health problems, and employee engagement. J. Occup. Organ. Psychol..

[28] Siegrist J. (1996). Adverse health effects of high-effort/low-reward conditions. J. Occup. Health Psychol..

[29] Siegrist J. (1996). Soziale Krisen und Gesundheit [Social Crisis and Health].

[30] Niedhammer I., Derouet-Gérault L., Bertrais S. (2022). Prospective associations between psychosocial work factors and self-reported health: Study of effect modification by gender, age, and occupation using the national French working conditions survey data. BMC Public Health.

[31] Kristensen T., Borritz M., Villadsen E., Christensen K.B. (2005). The Copenhagen Burnout Inventory: A new tool for the assessment of burnout. Work Stress.

[32] Borritz M., Rugulies R., Christensen K.B., Villadsen E., Kristensen T.S. (2006). Burnout as a predictor of self-reported sickness absence among human service workers: Prospective findings from three year follow up of the PUMA study. Occup. Environ. Med..

[33] Allen N.J., Meyer J.P. (1990). The measurement and antecedents of affective, continuance and normative commitment to the organization. J. Occup. Psychol..

[34] Meyer J.P., Allen N.J. (1991). A three-component conceptualization of organizational commitment. Hum. Resour. Manag. Rev..

[35] Choi B., Eum K., Kawakami N., Johnson J.V., Yim S., Paek D., Karasek R., Cho S.-i. Emotional Demand Items in the JCQ2.0 Korean Proto-Pilot Study. Proceedings of the 3rd ICOH International Conference on Psychosocial Factors at Work.

[36] Li J., Karasek R., Cho S.-i., Choi B., Johnson J.V., Ostry A., Landsbergis P. Reliability, Scale Structure, and Findings from Chinese JCQ2.0 Study. Proceedings of the 3rd ICOH International Conference on Psychosocial Factors at Work.

[37] Dollard M.F. Workplace psychosocial risks to mental health: National surveillance and the Australian Workplace Barometer project. Proceedings of the JCQ 2 Expert Workshop.

[38] Harkness J.A., Schoua-Glusberg A. (1998). Questionnaires in translation. ZUMA-Nachrichten Spez..

[39] Herdman M., Fox-Rushby J., Badia X. (1997). ‘Equivalence’ and the translation and adaptation of health-related quality of life questionnaires. Qual. Life Res..

[40] Bortz J., Döring N. (1995). Forschungsmethoden und Evaluation.

[41] Nunnally J.C., Bernstein I.H. (1994). Psychometric Theory.

[42] Meyer J.P., Allen N.J., Smith C.A. (1993). Commitment to organizations and occupations: Extension and test of a three-component conceptualization. J. Appl. Psychol..

[43] Baraldi A.N., Enders C.K. (2010). An introduction to modern missing data analyses. J. Sch. Psychol..

[44] Rubin D.B. (1976). Inference and missing data. Biometrika.

[45] Graham J.W., Cumsille P.E., Elek-Fisk E., Schinka J.A., Velicer W.F. (2003). Methods for handling missing data. Handbook of Psychology: Research Methods in Psychology.

[46] Schafer J.L., Graham J.W. (2002). Missing data: Our view of the state of the art. Psychol. Methods.

[47] Lüdtke O., Robitzsch A., Trautwein U., Köller O. (2007). Umgang mit fehlenden Werten in der psychologischen Forschung: Probleme und Lösungen. Psychol. Rundsch..

[48] SPSS Inc (2012). PASW® Missing Values 18.

[49] Kristensen T., Hannerz H., Hogh A., Borg V. (2005). The Copenhagen Psychosocial Questionnaire—A tool for the assessment and improvement of the psychosocial work environment. Scand. J. Work Environ. Health.

[50] Cohen J. (1977). Statistical Power Analysis for the Behavioral Sciences.

[51] SPSS Inc (2012). PASW Statistics 18.

[52] IBM (2012). SPSS Statistics 21.

[53] Aronsson G., Theorell T., Grape T., Hammarstrom A., Hogstedt C., Marteinsdottir I., Skoog I., Traskman-Bendz L., Hall C. (2017). A systematic review including meta-analysis of work environment and burnout symptoms. BMC Public Health.

